# CNAs’ Ratings of Nursing Home Residents’ Pain: The Role of CNA and Resident Traits

**DOI:** 10.1093/geroni/igaa057.1226

**Published:** 2020-12-16

**Authors:** Emily Behrens, Hyunjin Noh, A Lynn Snow, Patricia Parmelee

**Affiliations:** 1 The University of Alabama, Tuscaloosa, Alabama, United States; 2 Tuscaloosa VA Medical Center, Tuscaloosa, Alabama, United States; 3 University of Alabama, Tuscaloosa, Alabama, United States

## Abstract

Long-term care residents with and without cognitive impairment may experience undertreatment of persistent pain (Fain et. al, 2017). Certified nursing assistants (CNAs) are important sources of information about resident pain as they provide the majority of residents’ hands-on care. Therefore, assessing the accuracy of CNAs’ pain assessments and potential influencing factors may provide insight regarding the undertreatment of pain. Informed by prior research, this study examined resident pain catastrophizing and cognitive status as predictors of CNAs’ pain assessment accuracy. CNA empathy was examined as a moderating variable. Analyses confirmed a relationship between pain catastrophizing and CNA pain rating accuracy (R^2 = .205, p < .01), reflecting lower accuracy of ratings for residents higher in catastrophizing. Hypotheses predicting a relationship between resident cognitive status and CNA pain rating accuracy and moderating effects of empathy were disconfirmed. Challenges of conducting research in long-term care are discussed.

